# Novel Glial Cells Missing-2 (*GCM2*) variants in parathyroid disorders

**DOI:** 10.1530/EJE-21-0433

**Published:** 2022-01-13

**Authors:** Lucie Canaff, Vito Guarnieri, Yoojung Kim, Betty Y L Wong, Alexis Nolin-Lapalme, David E C Cole, Salvatore Minisola, Cristina Eller-Vainicher, Filomena Cetani, Andrea Repaci, Daniela Turchetti, Sabrina Corbetta, Alfredo Scillitani, David Goltzman

**Affiliations:** 1 Metabolic Complications and Disorders, Research Institute-McGill University Health Centre, Montreal, Quebec, Canada; 2 Division of Medical Genetics and Unit of Endocrinology, Fondazione IRCCS Casa Sollievo della Sofferenza, San Giovanni Rotondo, Italy; 3 Department of Laboratory Medicine and Pathobiology, University of Toronto, Toronto, Ontario, Canada; 4 Department of Internal Medicine and Medical Disciplines, ‘Sapienza’ Rome University, Rome, Italy; 5 Department of Medical Sciences and Community, Fondazione Ca’Granda IRCCS Ospedale Maggiore Policlinico, Milan, Italy; 6 University Hospital of Pisa, Pisa, Italy; 7 Unit of Endocrinology, S. Orsola Malpighi Hospital, Bologna, Italy; 8 Center for the Studies of Hereditary Cancers, Department of Medical and Surgical Sciences, University of Bologna, Bologna, Italy; 9 Endocrinology and Diabetology Service, IRCCS Istituto Ortopedico Galeazzi, Milan, Italy; 10 Department of Biomedical, Surgical and Dental Sciences, University of Milan, Milan, Italy

## Abstract

**Objective:**

The aim of this study was to analyze variants of the gene glial cells missing-2 (*GCM2*), encoding a parathyroid cell-specific transcription factor, in familial hypoparathyroidism and in familial isolated hyperparathyroidism (FIHP) without and with parathyroid carcinoma.

**Design:**

We characterized 2 families with hypoparathyroidism and 19 with FIHP in which we examined the mechanism of action of *GCM2* variants.

**Methods:**

Leukocyte DNA of hypoparathyroid individuals was Sanger sequenced for *CASR, PTH, GNA11* and *GCM2* mutations. DNA of hyperparathyroid individuals underwent* MEN1, CDKN1B, CDC73,* *CASR*, *RET* and *GCM2* sequencing. The actions of identified* GCM2* variants were evaluated by *in vitro* functional analyses.

**Results:**

A novel homozygous p.R67C *GCM2* mutation which failed to stimulate transcriptional activity in a luciferase assay was identified in affected members of two hypoparathyroid families. Oligonucleotide pull-down assay and *in silico* structural modeling indicated that this mutant had lost the ability to bind the consensus GCM recognition sequence of DNA. Two novel (p.I383M and p.T386S) and one previously reported (p.Y394S) heterozygous *GCM2* variants that lie within a C-terminal conserved inhibitory domain were identified in three affected individuals of the hyperparathyroid families. One family member, heterozygous for p.I138M, had parathyroid carcinoma (PC), and a heterozygous p.V382M variant was found in another patient affected by sporadic PC. These variants exerted significantly enhanced *in vitro*transcriptional activity, including increased stimulation of the PTH promoter.

**Conclusions:**

We provide evidence that two novel *GCM2* R67C inactivating mutations with an inability to bind DNA are causative of hypoparathyroidism. Additionally, we provide evidence that two novel GCM2 variants increased transactivation of the PTH promoter *in vitro* and are associated with FIHP. Furthermore, our studies suggest that activating GCM2 variants may contribute to facilitating more aggressive parathyroid disease.

## Introduction

**Figure 1:**
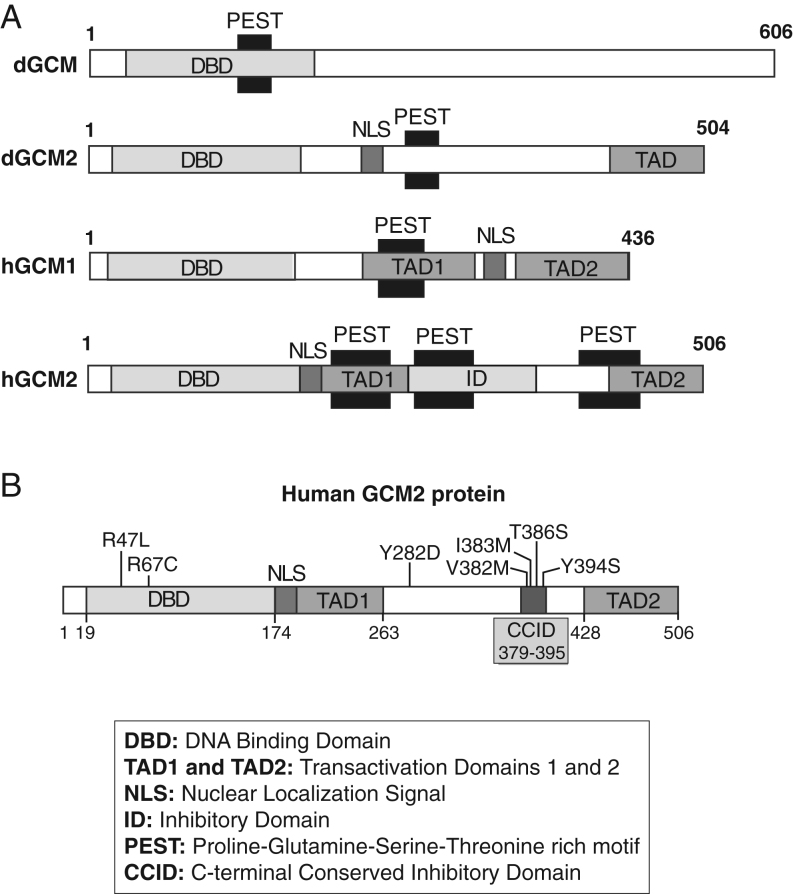
Schematic representation of GCM protein domains and position of mutations/variants on human GCM2. (A) Protein structures of *Drosophila* and human GCM proteins are shown. All proteins possess a DNA-binding domain (DBD), nuclear localization signal (NLS), one or two transactivating domain 67 (TAD) and proline (P), glutamic acid (E), serine (S), and threonine (T)-rich (PEST) domain(s) implicated in rapid protein turnover. Human GCM2 contains an inhibitory domain (ID) and a unique C-terminal conserved inhibitory domain (CCID) (B). Positions of missense mutations in human GCM2 are shown. Germline mutations associated with hypoparathyroidism, and within the DBD, R67C (novel) and previously identify R47L (18). Germline mutations/variants associated with hyperparathyroidism, Y282D (32) and within the CCID, V382M, I383M, T386S and Y394S (14; present publication) are shown.

Glial cells missing 2 (GCM2) belongs to a small family of nuclear transcription factors involved in fundamental developmental processes (1, 2). Initially identified in *Drosophila* (3), the functions of the two mammalian orthologs, *GCM1*/*GCMA* and *GCM2*/*GCMB,*have diverged significantly from those in the fly (4, 5). GCM family members share an evolutionary highly conserved zinc-coordinating DNA-binding domain (DBD) called the GCM motif in their amino-terminus that recognizes an octameric nucleotide sequence (6, 7). The carboxyl-terminal portion of GCM1 and GCM2 is poorly conserved and includes a nuclear localization signal (NLS), one or two transcriptional activation domains (TAD1 and TAD2) and potential PEST sequences found in proteins displaying rapid turnover (Fig. 1A) (8).

*Gcm2*-null mice fail to develop parathyroid glands, leading to primary hypoparathyroidism (9, 10, 11). Human *GCM2* is located on chromosome 6p24.2 and expressed exclusively in the developing and mature parathyroid hormone (PTH)-secreting cells of the parathyroid glands (12).

Primary hypoparathyroidism encompasses a heterogeneous group of disorders in which hypocalcemia and hyperphosphatemia occur as a result of deficient PTH secretion (13, 14). Familial isolated hypoparathyroidism (FIH) shows multiple modes of inheritance. Autosomal dominant inheritance occurs with heterozygous inactivating mutations of the *GCM2, PTH* or* TBX1* genes (MIM #146200, #617343, #602054) (15, 16, 17) or with heterozygous activating mutations of the *calcium-sensing receptor* (*CASR*) or the *G protein α11 subunit* (*GNA11*) genes (MIM# 601198, 615361) (18, 19, 20). Alternatively, autosomal recessive FIH may be seen with homozygous inactivating mutations of the *GCM2, PTH* or *AIRE* genes (MIM #146200, 618883, 607358) (16, 21, 22, 23). In the present study, we examined *GCM2* mutations in two families presenting with hypoparathyroidism.

*GCM2* variants have also been associated with primary hyperparathyroidism (PHPT) (24). PHPT is the most common cause of hypercalcemia and most cases are caused by a solitary, sporadic (non-familial) parathyroid adenoma (25, 26, 27). Approximately 5–10% of PHPT is hereditary. Familial isolated hyperparathyroidism (FIHP) (MIM #145000) has been defined as hereditary PHPT without the association of other disease or tumors (28). A small proportion of these cases is caused by variants of other monogenic diseases – multiple endocrine neoplasia (MEN) type 1, 2A or 4 (MEN1, MEN2A, MEN4), hyperparathyroidism jaw tumor syndrome (HPT-JT) or familial hypocalciuric hypercalcemia – but additional genes are likely involved in the other cases (29).

*GCM2* expression was reported to be upregulated in abnormal parathyroid glands of hyperparathyroidism (30), and *GCM2-*activating variants have been proposed as candidate predisposition alleles in sporadic parathyroid tumors. A *GCM2* variant, p.V382M, was identified in 30 parathyroid adenomas (31) and clustered in a small domain of 17 amino acids termed as the ‘C-terminal conserved inhibitory domain (CCID)’ (24) (Fig. 1B). Another variant, Y282D, was found in a large cohort of Italian PHPT patients and in two smaller replication cohorts (32). *GCM2* variants have also been found in 396 sporadic parathyroid adenomas with a frequency of activating GCM2 CCID (p.V382M and p.Y394S) and Y282D variants of 1.52 and 5.05%, respectively (33). Functional studies have shown that Y282D was transcriptionally more active (32, 34) or similar to (24) the WT. GCM2 heterozygous gain-of-function variants have also been reported in FIHP (31, 35) with a frequency of 18%. In contrast, Correa *et al*. (36) observed a downregulation of GCM2 mRNA levels in adenomas of PHPT. Consequently, there is no universal consensus on the causal role of GCM2 in PHPT. In the present study, we identified heterozygous novel and recurrent variants of GCM2 in the probands of FIHP kindreds that were negative for mutations in the *MEN1*, *CDKN1B, CDC73, RET* or *CASR* genes. Functional analyses of these *GCM2* variants were performed.

## Subjects and methods

### Subjects

The Institutional Research Ethics Boards of the University of Toronto and the Fondazione IRCCS Casa Sollievo Della Sofferenza Hospital approved the protocol, and informed consent was obtained from the proband and family members.

**Figure 2:**
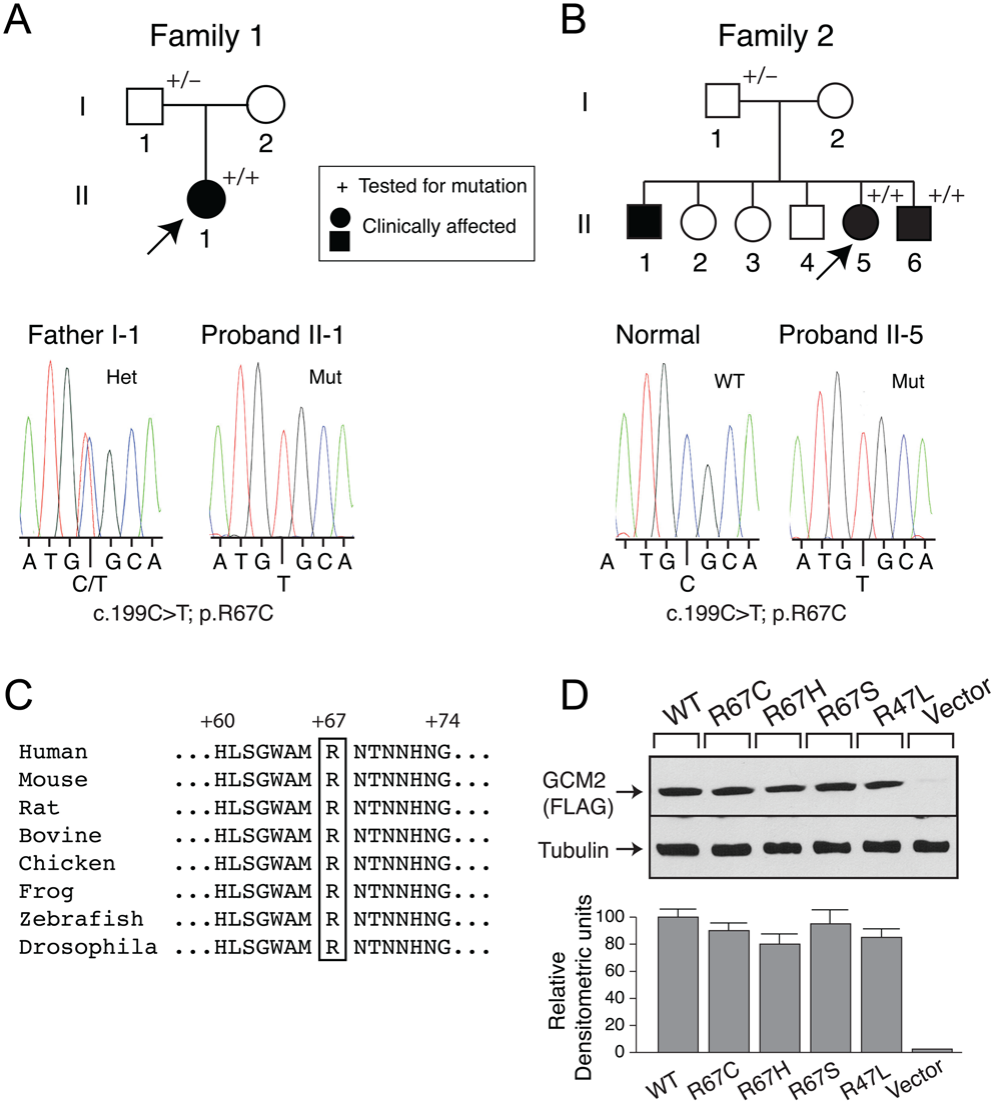
Detection of a *GCM2* mutation in two kindreds with autosomal recessive hypoparathyroidism. Pedigree structure (top panels) and sequence chromatograms (lower panels) of (A) family 1 and (B) family 2. Clinical status is indicated by open symbols (unaffected) and solid symbols (affected). Probands are indicated by the arrow. The presence (+) or absence (−) of a mutated *GCM2* allele in tested family members is shown. (A) Direct sequence analysis of the exon 2 genomic DNA amplicon of the proband, individual II-1 (right) revealed a homozygous missense mutation, compared with a heterozygous missense mutation in the father, individual I-1 (left). (B) Direct sequence analysis of the exon 2 genomic DNA amplicon of the proband, individual II-5 (right) revealed a homozygous missense mutation, compared with an unrelated normal individual (left). (C) The GCM2 protein sequences from diverse species were aligned as described in Subjects and Methods. The residue corresponding to the human GCM2 arginine 67 mutation is boxed. (D) Expression of WT and mutant GCM2 proteins. Western blot analysis (top panel) of extracts of HEK293 cells that had been transfected with FLAG-tagged WT or mutant R67 constructs. β-tubulin was the loading control. Densitometric analysis of Western blot was performed (lower panel). A full color version of this figure is available at https://doi.org/10.1530/EJE-21-0433.

FIH was diagnosed in individuals within each family with hypocalcemia, low or undetectable serum PTH concentrations and absence of other clinical manifestations of syndromic hypoparathyroidism. The two hypoparathyroid probands in whom FIH was diagnosed with *GCM2* mutations were sick infants who were brought into the emergency room by parents and were found to be severely hypocalcemic. The brother of the proband in family 2 also had severe hypocalcemia with seizures and a *GCM2* mutation. The asymptomatic fathers of the probands in the two families were both heterozygous for the *GCM2* mutations (Fig. 2A and  B). Mutation screening for *PTH*, *CASR*, as well as *GCM2* were performed in the probands and in affected or unaffected family members from whom DNA could be obtained. No germline mutations in *CASR* and *PTH* exons were found.

FIHP was diagnosed in individuals within each family by the presence of hypercalcemia, inappropriately normal or elevated serum PTH concentrations, no evidence of clinical manifestations of familial syndromic hyperparathyroidism and no germline mutations in *MEN1, CDKN1B, CDC73, RET, GNA11* or *CASR* genes. The 19 Italian FIHP kindreds were from Endocrine Clinics in San Giovanni Rotondo, Rome, Pisa and Milan. This report includes all FIHP kindreds consecutively studied and followed up in the past 10 years (Supplementary Table 1, see section on supplementary materials given at the end of this article). All are Caucasians. One case of parathyroid carcinoma was identified during the evaluation of a family for FIHP. Subsequently, 18 cases that presented with generally severe hypercalcemia, and which were evaluated for hyperparathyroidism and found to have parathyroid carcinoma, were also studied (Supplementary Table 2). Surgical neck exploration was performed in each of these patients and suggested local invasion or regional metastasis, and a diagnosis of parathyroid carcinoma was established by histopathologic assessment of resected parathyroid tissue.

### DNA sequence analysis

Written informed consent was obtained for blood collection and analysis of relevant genes in the patients and relatives. Patient leukocyte DNA was isolated using standard methods (Qiagen). DNA Sanger sequence analysis of protein-coding exons and adjacent splice sites of the *RET*, *GNA11*, *PTH*, *MEN1*, *CDKN1B, CDC73*, *CASR* and the *GCM2* genes was conducted as described previously (37). Supplementary Table 2 lists the primers used for gene sequencing and mutagenesis.

Publicly accessible databases were examined for the presence of the identified sequence variants, including: *e!Ensembl* (http://uswest.ensembl.org)(for access to 1000 genomes), the Genome Aggregation Database (gnomAD) (http://gnomad.broadinstitute.org/) representing 277,238 alleles from ExAc and other sources and ClinVar (https://www.ncbi.nlm.nih.gov/clinvar/) an archive of reports of the relationships among human variations and phenotypes.

### Protein sequence alignment and three-dimensional (3D) homology modeling of GCM2 DNA-binding domain structure

Evolutionary conservation and predicted functional effects of the variant were assessed *in silico* with Polymorphism Phenotype version 2 (Polyphen-2: http://genetics.bwh.harvard.edu/pph2/) and Sorting Intolerant from Intolerant (SIFT: http://sift.jcvi.org/).

Homology of human, other vertebrate and invertebrate GCM2 orthologs was assessed using the online Clustal Omega tool (https://www.ebi.ac.uk/Tools/msa/clustalo/).

A 3D structure of the human GCM2 DNA-binding model was generated based on the crystal structure of the murine GCM1 DBD in complex with DNA ((38); PDB ID: IODH) using USCF Chimera (39) and Modeller (40) softwares.

### Plasmid construction and site-directed mutagenesis

Human WT GCM2 cDNA (Origene Technologies, Rockville, MD) was subcloned into pCMV-Tag 2A (Stratagene) to generate an in-frame NH_2_-terminus FLAG-tag construct. Mutations were introduced using the QuikChange Site-Directed Mutagenesis kit (Agilent). The correctness of the constructs was verified by sequencing.

Akiyama *et al.* (6) identified a consensus GCM-binding element, 5’-ATGCGGGT-3’, as the optimal recognition sequence for GCM1 and GCM2. The 3xgbs-lux reporter construct in which multimerized GCM elements (7) were cloned upstream of the SV40 promoter in the pGL3-Promoter vector (Promega) was described previously (18).

The human PTH promoter was created according to Kawahara *et al.* (41). Region −1200/+54 (+1, designating the transcription start site) containing a GCM-responsive element at −390/−383 was amplified from human genomic DNA and cloned into pGL3-basic promoter vector (Promega).

### Cell culture, transfection and Western blot analysis

HEK293 cells were cultured in Dulbecco’s modified Eagle’s medium supplemented with 10% FBS (Wisent, St-Bruno, Qc, Canada). Transfections were performed using Polyfect (Qiagen). Ten micrograms of protein cell lysate containing protease inhibitors (Roche) were run on sodium dodecyl sulfate (SDS)-PAGE , followed by Western blotting. Membranes were probed with polyclonal anti-Flag antibody (Sigma–Aldrich) for GCM2 expression and with β-tubulin.

### Luciferase reporter assay

For promoter/reporter studies, HEK293 cells were grown in six-well plates to 70% confluence and transfected with pGL3 (vector alone) or 3xgbs-lux (or PTH promoter) constructs with WT or mutant GCM2 constructs (or pCMVTag2 as empty vector control). β-galactosidase vector (internal control) was also transfected. After 48 h, cells were lysed, the supernatant luciferase activity was measured and normalized to β-galactosidase activity as described (18).

### Oligonucleotide precipitation assay

HEK293 cells were transfected with pCMVtag2, WT or mutant Flag-GCM2 constructs. After 48 h, cells were lysed by sonication in cold HKMG buffer (10 mM HEPES, pH 7.9, 100 mM KCl, 5 mM MgCl2, 10% glycerol, 1 mM dithiothreitol and 0.1% Nonidet P-40) containing protease inhibitors (Roche) and spun (5 min, 10 000 ***g***, 4°C) to remove cell debris. One mg of proteins was incubated for 16 h with rotation at 4°C with 1 μg of double-stranded biotinylated oligonucleotides, corresponding to the WT or mutated consensus GCM element (16). Anti-Flag beads (Sigma–Aldrich) were added and rotated for 5–6 h at 4°C followed by centrifugation at 1500 ***g*** for 1 min at 4°C. Pelleted beads were washed and resuspended in HKMG buffer. Avidin-Horse radish peroxidase (HRP) secondary antibody was added, the sample was incubated with rotation at 4°C for 1 h and then centrifuged at 1500 ***g*** for 5 min at 4°C. The pellet was washed, resuspended in PBS and HRP substrate and tetramethylbenzidine (TMB; Sigma–Aldrich) was added. Beads were spun at 1500 ***g*** and the absorbance at 605 nm of the supernatant was measured.

### Statistical analysis

The data are expressed as mean ± s.e.m. of triplicate estimations with each experiment repeated three times. A *P* value <0.05 is considered statistically significant. Difference between WT and variants were assessed using either *t*-tests or ANOVA with Bonferroni corrections.

## Results

### Part 1 *GCM2* mutation in two families with autosomal recessive hypoparathyroidism

#### Phenotypic characterization of hypoparathyroid family members

##### Family 1

The proband, an infant boy (Fig. 2A, individual II-1), exhibited hypocalcemic hypoparathyroidism and had normocalcemic parents (individuals I-1 and I-2). At 11 days of life, the proband presented with hypocalcemic seizures. His initial blood examination showed: total serum calcium (tCa^2+^), 1.59 nmol/L (NR = 2.2–2.6); serum ionized calcium (iCa^2+^), 0.75 mmol/L (NR = 1.13–1.32); serum magnesium, 0.68 mmol/L (NR = 0.7–1.2); serum PTH, 4 ng/L (NR = 10–60); serum 25-hydroxyvitamin D (25(OH)D), 41 nmol/L (N > 50); spot urine calcium to creatinine ratio, 1.2 mmol/mmol (N < 1.5). Fluorescence *in situ* hybridization analysis demonstrated two intact copies of the 22q11.2 locus, thus excluding DiGeorge syndrome. Treatment began with 50 mg/kg/day elemental calcium as calcium carbonate, alphacalcidol (1α-OH-vitamin D_3_), 0.08 μg/kg/day and cholecalciferol (vitamin D_3_). At 6 weeks of age, the infant presented with further hypocalcemic seizures, with serum tCa^2+^, 1.79 mmol/L; serum iCa^2+^, 0.89 mmol/L; 25(OH)D, 95 nmol/L; PTH, 2 ng/L; urine calcium/creatinine ratio, 0.85. The admission was complicated by local skin irritation secondary to IV calcium infusion. With increasing serum calcium concentration, there was a marked increase in urinary calcium excretion (urine calcium/creatinine ratio, 6.23), and therefore, a thiazide diuretic was added. Over the next 2 months, increasing doses of medication were required, but the s.c. irritation resolved almost completely. At 4 months of age, laboratory evaluation showed: serum tCa^2+^, 1.87 mmol/L; serum iCa^2+^, 0.94 mmol/L; serum phosphate, 3.19 mmol/L (NR = 1.5–2.1); serum creatinine, normal; serum 25(OH)D, 148 nmol/L; urinary calcium/creatinine ratio, 0.7 mmol/mmol. Medication at the time included 1 μg alfacalcidol TID, 0.4 μg/kg/day; 100 mg/kg/day elemental calcium as calcium carbonate, hydrochlorothiazide, 4 mg/kg/day. From 6 months of age, serum Ca^2+^ levels improved, and medication was progressively decreased. He was most recently taking 0.5 μg alfacalcidol BID, 0.07 μg/kg/day and calcium carbonate, 200 mg BID. Hydrochlorothiazide was withdrawn. The family was subsequently lost to clinical follow-up.

##### Family 2

Three siblings (Fig. 2B, individuals II-1, II-5, II-6) presented in infancy, with severe hypoparathyroidism and hypocalcemia, two of whom then underwent genotyping (individuals II-5, II-6). Parents (individuals I-1 and I-2) were both normocalcemic. The family was subsequently lost to clinical follow-up.

#### Identification of a *GCM2* mutation

*CASR* and *PTH* exons (including exon/intron boundaries) were normal in all samples.

*GCM2* sequence analysis of the family 1 proband (II-1) (Fig. 2A) identified a novel homozygous c.199C>T; p.R67C variant encoded by exon 2. The father was heterozygous for the mutation.

The same homozygous variant was found in individuals II-5 and II-6 of family 2 (Fig. 2B). The father was also heterozygous for the mutation.

In view of the common ancestry (Somalia) of the two families, it is likely that there was a founder effect.

Arginine 67 is located in the DBD (Fig. 1). Protein alignment of different GCM2 species shows that this arginine is highly conserved from human to *Drosophila* (Fig. 2C). *In silico* analysis suggested the R67C to be probably damaging (Polyphen) and deleterious (SIFT) (Table 1). R67C does not appear in any of the public databases but two variants, R67S and R67H, have been reported in 1000 genomes and gnomAD databases.

**Table 1:** R67 variants in public databases.^a,b,c,d^

Nucleotide change	Protein change	Variant ID (rs#)	1000 genomes	gnomAD	Polyphen	SIFT
c.199C>T^d^	p.R67C	–	–	–	Probably damaging	Deleterious
c.199C>A	p.R67S	532834782	1 AFR (ACB)	1 AFR		
			Allele frequency: 1/5008 (0.000199680)	Allele frequency: 1/251458 (0.000003977)	Probably damaging	Deleterious
c.200G>A	p.R67H	200953294	1 EAS (CHS)	1 EAS		
			Allele frequency: 1/5008 (0.000199680)	Allele frequency: 1/251458 (0.000003977)	Probably damaging	Deleterious

^a^All variants are heterozygotes; ^b^reference sequences: NM_004752.4 (transcript), NP_004743.1 (protein); ^c^No entry in ClinVar and HGMD (The Human Gene Mutation Database); ^d^c.199C>T is in linkage with c.-44T>C (rs16870746) and c.457-55A>G (rs145588606).

ACB, African Caribbean in Barbados; AFR, African, African–American; CHS, Southern Han Chinese; EAS, East Asian; gnomAD, Genome Aggregation Database.

#### Expression and transcriptional activities of WT and Arg67 variants

In addition to R67C, we also engineered and tested variants R67H and R67S for expression and functionality. Western blot analysis of Flag-tagged-GCM2 WT and variants revealed a protein band of expected size (~56 kDa). No immunoreactivity was observed in lysates from cells transfected with vector alone (Fig. 2D). Densitometric analysis showed equivalent levels of expression (relative to housekeeping protein β-tubulin) for GCM2 WT and variants. Therefore, none of the amino acid substitutions appears to affect protein stability.

**Figure 3:**
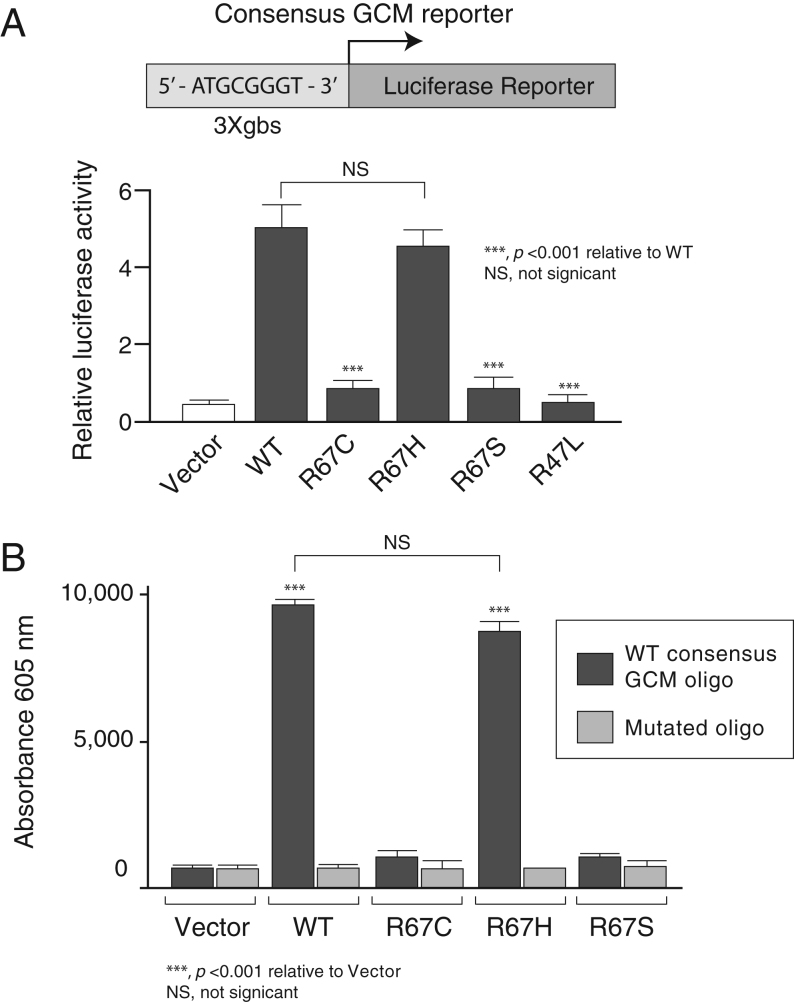
Transcriptional activities and DNA binding of WT and mutant GCM2 proteins with a synthetic GCM responsive promoter (3X-gbs). (A) Promoter–reporter construct containing three consensus GCM response elements in tandem upstream of a luciferase reporter gene (3xgbs-lux) was transfected into HEK293 cells with vector, WT or mutants (R67C, R67H, R67S or R47L). After 48 h, cells were harvested and luciferase activity of extracts measured. Values are mean ± s.e.m. of six estimations. (B) R67C mutant loses ability to bind to DNA. Biotinylated oligonucleotide pull-down assays were conducted on extracts of HEK293 cells that had been transfected 48 h before harvesting with either empty vector alone or Flag-GCM2 WT or mutants. The oligonucleotides used represented the WT and mutant GCM consensus element.

Several missense mutations of GCM2 reported in FIH kindreds caused lower or no transcriptional activity in luciferase assays (16, 21). To assess transcriptional activities, WT and R67 variant constructs were co-transfected with the synthetic 3xgbs-lux construct that has three consensus GCM response elements in tandem driving the luciferase reporter gene. When transfected with control empty expression vector (pCMV-tag2), 3xgbs-lux exhibited basal activity but co-transfection with WT GCM2 elicited a 4.4-fold increase in transcriptional activity relative to vector alone (Fig. 3A). Co-transfection of R67C, the variant found in our families, and R67S resulted in only basal levels of activity, but R67H showed potency similar to that of WT GCM2. As a negative control, a previously described inactivating mutant, R47L (16) only displayed basal activity.

#### R67C mutant does not bind DNA

Since arginine 67 is located within the DBD, we speculated that the R67C mutation can disrupt the formation of the GCM2:DNA complex, and therefore, we performed *in vitro* and *in silico* experiments to examine this hypothesis. We conducted oligonucleotide precipitation assays with double-stranded biotinylated oligonucleotides corresponding to the WT or mutated consensus *GCM* element (Fig. 3B). Lysates from HEK293 cells transfected with empty vector, WT or variant R67 variants served as a protein source. Binding of WT and R67H proteins to the consensus oligo (but not the mutated one) was detected, consistent with their luciferase activity observed in the promoter assay. In contrast, transcriptionally inactive R67C and R67S demonstrated no binding to the *GCM* consensus oligo.

**Figure 4:**
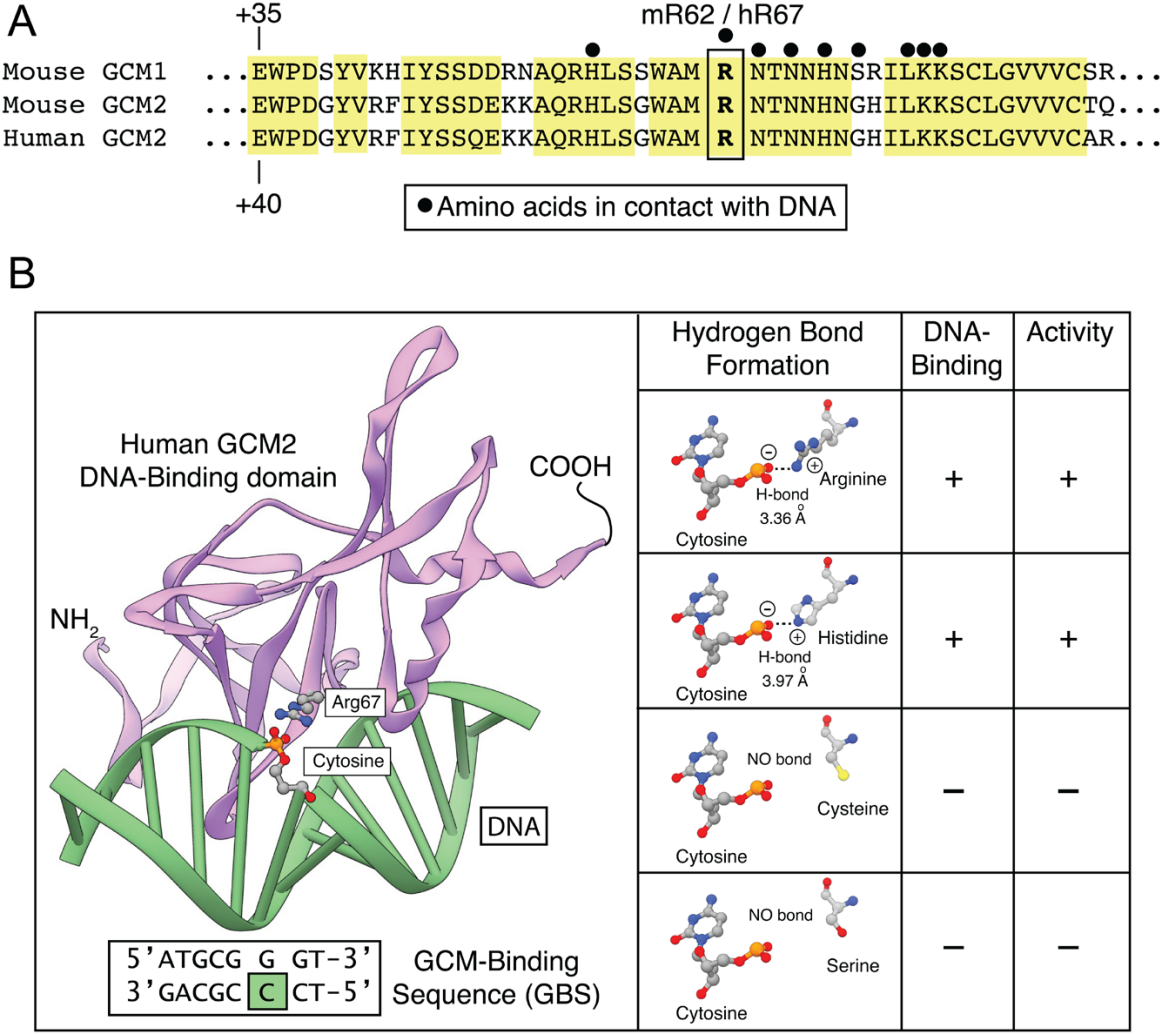
Detailed analysis of the structural effect of R67 variants. A. Alignment of mouse and human GCM1 and GCM2. Conserved residues and conservatively substituted residues are drawn on a yellow background. Black dots (•) indicate DNA-contacting residues. Murine GCM1 arginine 62 and murine and human GCM2 arginine 67 are boxed. (B) Three-dimensional model of the GCM2 DNA-binding domain in complex with DNA. Ribbon representation of the GCM motif (pink) bound to its cognate DNA (green). The residue arginine 67 is shown in ball and stick presentation as is the DNA cytosine that it makes contact with. Expanded view of the structure of R67 and the substitutions: R67, H67, C67 and S67 shown in ball and stick format. In R67 and H67, the normal positive sidechain (oxygen atoms in blue) and negative DNA cytosine (hydrogen atoms in red) can form a hydrogen bond. In the presence of C67 or S67, the side chains cannot contribute to the formation of a hydrogen bond because they do not provide a positive charge. A full color version of this figure is available at https://doi.org/10.1530/EJE-21-0433.

The crystal structure of the DBD of murine GCM1 bound to a 13 bp DNA duplex containing its consensus target site has been reported (PDB# 1ODH) (38). DBDs of murine GCM and human GCM2 share more than 85% homology and 68% sequence identity allowing the generation of a reliable 3D structure by comparative modeling. Alignment of GCM1 and GCM2 shows that all amino acids identified as being important for mGCM1 DNA binding are fully conserved (Fig. 4A). Arginine 67 of human and mouse GCM2 is equivalent to murine GCM1 arginine 62 which has been shown to be in direct contact with a cytosine at position 6 in the DNA of the GCM consensus motif (38). Using Modeller and Chimera, we generated a 3D model of human GCM2 DBD:DNA complex (Fig. 4B). The model shows that the distance between the positive charge of arginine 67 and the negative phosphate charge of the cytosine of the DNA backbone is 3.36 Å, allowing the formation of a hydrogen bond. Mutating arginine 67 to either serine or cysteine does not lead to any hydrogen bonding with cytosine 6 because the side chains of these amino acids are not positively charged. From our *in vitro* assays, we know that the variant 67H has transcriptional activity and DNA-binding capacity similar to WT GCM2. To verify if this was structurally valid, we mutated arginine 67 in our model and moved the amino acid around the axis using the rotamer tool to allow hydrogen bond formation with approximately the same distance as WT. After correction for clashes, a rotamer with an approximate distance of 3.97 Å between the positively charged imidazolium ring of histidine 67 to the nearest oxygen of the DNA backbone was found. This gave crystallographic support for the WT behavior of the histidine variant.

### Part 2 *GCM2* variants in families with familial isolated hyperparathyroidism and sporadic parathyroid carcinoma

#### Phenotypic characterization of hyperparathyroid family members and identification of variants

**Figure 5:**
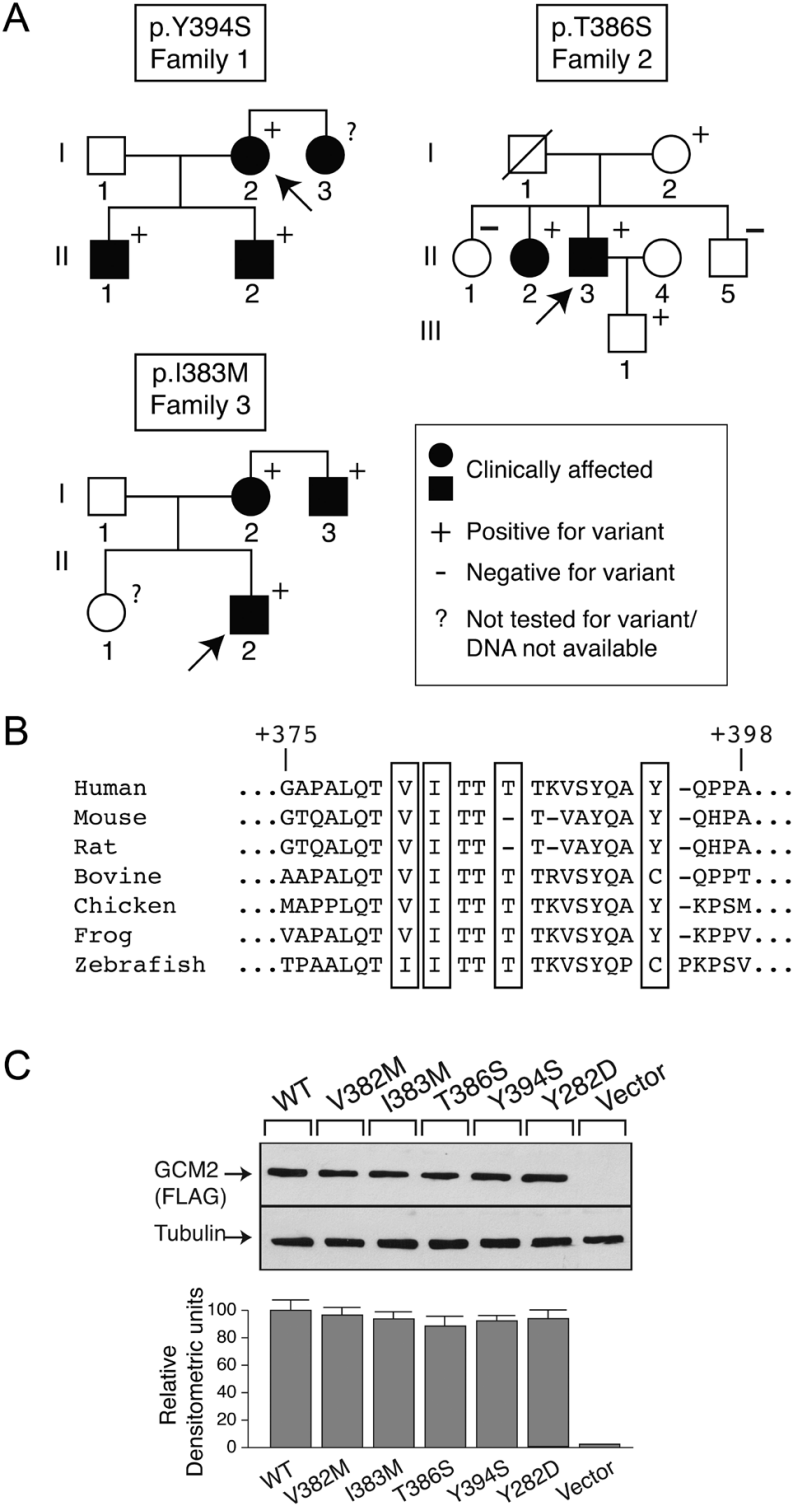
Detection of *GCM2* variants in three kindreds with FIHP. (A) Pedigrees: clinical status is indicated by open symbols (unaffected) and solid symbols (affected). Proband is indicated by the arrow. The presence (+) or absence (−) of a *GCM2* variant allele in tested family members is shown. Heterozygous c.1181A>C; p.Y394S (recurrent, Family 1); c.1156A>T; p.T386S (novel, Family 2); c.1149C>G; p.I383M (novel, Family 3). (B) The GCM2 protein sequences from diverse species were aligned as described in Materials and Methods. (C) Expression of WT and variant GCM2 proteins. Western blot analysis (top panel) of extracts of HEK293 cells that had been transfected with FLAG-tagged wild-type, or GCM2 variant constructs. β-tubulin was the loading control. Densitometric analysis of Western blot (lower panel).

Nineteen hyperparathyroid FIHP kindreds from Endocrine Clinics in San Giovanni Rotondo, Rome, Pisa and Milan were studied. Individuals were diagnosed with PHPT if they presented with hypercalcemia and elevated or inappropriately normal circulating PTH (Table 2). In all families except two, at least one family member with PHPT had histopathologically verified abnormal parathyroid tissue. None of the probands had germline mutations in *MEN1*, *CDKN1B, CDC73*, *RET, GNA11*or *CASR* genes. Figure 5A shows the pedigree of three families where *GCM2* heterozygous changes within the CCID region (24) were identified. One was previously reported: c.1181A>C; p.Y394S (family 1) and two were novel: c.1156A>T; p.T386S (family 2) and c.1149C>G; p.I383M (family 3). The biochemical and clinical features of these families are shown in Table 2. Supplementary Table 1 reports the severity of the clinical manifestations in the patients.

**Table 2:** Biochemistry of FIHP kindreds.

Variant	Kindred/pedigree	Age at diagnosis of PHPT (years)	Presence of variant^a^	Total calcium (mmol/L)^†^	Ionized Ca^2+^(mmol/L)	Phosphate (mg/dL)^†^	PTH (pg/mL)^†^	Operations, *n*	Pathology	Glands resected, *n*
Y394S	1									
	Proband (I-2)	62	Yes	2.62	1.37	3.2	62	ND	–	–
	Son (II-1)	44	Yes	2.65	1.33	2.9	43.2	–	–	–
	Son (II-2)	43	Yes	2.55	1.32	2.83	43.5	–	–	–
T386S	2									
	Proband (II-3)	58	Yes	3.7	2.25	2.2	113	1	Hyperplasia	3 (largest: 1.8 cm)
	Mother (I-2)	90*	Yes	2.23	n/a^c^	3.7	62	–	–	–
	Sister (II-1)	65	No	2.27	n/a	3.2	56	–	–	–
	Sister (II-2)	69*	Yes	2.99	1.56	2.9	104	1	Hyperplasia	4 (largest: 2.6 cm)
	Brother (II-5)	67*	No	2.37	n/a	n/a	51	–	–	–
	Son (III-1)	29*	Yes	2.4	1.22	3.2	58	–	–	–
I383M	3									
	Proband (II-1)	38	Yes	3.29	n/a	2.0	230	2	Carcinoma and hyperplasia	3 (largest: 3.5)
	Mother (I-2)	67	Yes	2.57	n/a	2.6	95	1	Adenomas	2 (largest: 1 cm
	Uncle (II-3)	55	Yes	3.24	n/a	1.9	841	2	Adenomas and atypical adenoma	3 (largest: 4.5 cm)
	Sister (II-2)	42*	?	2.1	n/a	2.76	30	–	–	–

^a^when present, the variant is a heterozygous change; *Age at recruitment; ^†^Normal rage for total calcium: 2.05–2.50 mmol/L; ionized Ca^2+^: 1.17–1.31 mmol/L; phosphate: 2.7–4.5 mg/dL; PTH: 10–65 pg/mL.

ND, not yet operated.

##### Family 1 (p.Y394S)

The proband (Fig. 5A, I-2) was a 62-year-old female who presented with nausea and fatigue and was found to be hypercalcemic and hypophosphatemic with an elevated PTH level. (Supplementary Table 1). A neck ultrasound revealed two lesions outside the thyroid capsule and behind the left and right lobes of the thyroid gland consistent with parathyroid nodules. Surgical treatment in the proband was scheduled but postponed. The proband`s sister (Fig. 5A, I-3) underwent surgery for PHPT, and a parathyroid adenoma confirmed by pathology was removed, suggesting familial disease. However, DNA could not be obtained from the sister. Consequently, the proband`s sons (Fig. 5A, II-1 and II-2) were studied. After the diagnosis of hyperparathyroidism was established in the sons and other known genetic PHPT forms were excluded (by hormonal and genetic analyses), screening of *GCM2* confirmed the presence of the p.Y394S variant (Fig. 5A).

##### Family 2 (p.T386S)

The proband (Fig. 5A, II-3), a 58-year-old male, was admitted to the hospital in 2016 for progressive fatigue and polyuria. Serum biochemistry showed high calcium and PTH levels along with hypercalciuria and high creatinine. After hydration, with consequent reduction of serum creatinine and calcium levels, and subsequent i.v. infusion of zoledronic acid 4 mg, the patient underwent surgical excision of three parathyroid glands diagnosed as hyperplastic by pathological examination. Since then, his serum calcium has been in the normal range during follow-up. In 2017, his sister (Fig. 5A, II-2), aged 69, presented with the same symptoms, clinical and biochemical picture. At surgery, four hyperplastic parathyroid glands were removed. After almost 4 years of follow-up, her serum calcium has been normal.

##### Family 3 (p.I383M)

The proband (Fig. 5A, II-2), a 38-year-old male, had surgery twice in March 2014. Initially, he underwent right inferior parathyroidectomy and right thyroid lobectomy. A parathyroid carcinoma (PC) was diagnosed based on the histological picture of invasion of the capsule and into the surrounding structures as well as the invasion of perineural spaces and of perithyroidal muscles. Because of persistent high serum calcium levels, he was reoperated after 3 weeks and two enlarged parathyroid glands were excised and diagnosed by the pathologist as hyperplastic. He was hypocalcemic post-surgery for some months but has been normocalcemic up to his last visit in July 2020. The proband’s uncle, aged 55, (Fig. 5A, I-3) had two parathyroid adenomas (left and right inferior glands) removed in 2009. Due to persistent hypercalcemia, he had surgery again in 2011 for the excision of an atypical parathyroid adenoma. Since then, his serum calcium level has remained normal (last visit in May 2020). The proband’s 67-year-old mother (Fig. 5A, I-2) received a diagnosis of PHPT in 2012, and in 2015, her left and right inferior parathyroid glands were excised. A pathological diagnosis of parathyroid adenomas was made. She has been normocalcemic up to her last follow-up of July 2020.

Since the proband of family 3 had a PC, we sought evidence that GCM2 variants could be identified in other patients with a diagnosis of PC but negative for germline and somatic CDC73 mutation. Thus, in an additional 16 sporadic PC not bearing CDC73 mutations, we searched for germline GCM2 mutations, and in 13, we also searched for somatic mutations. One patient out of the 16 with carcinomas had a germline p.V382M variation. This patient had a PC which was diagnosed post-surgery based on the histological analysis of the removed enlarged parathyroid which showed multiple figures of invasion of the capsule with infiltration of surrounding fibro-adipose tissue. None of the 13 PCs had a somatic mutation in GCM2.

Figure 5B shows the alignment of the conserved CCID domain. Publicly accessible databases were examined for the presence of the activating variants (Table 3). The two novel variants I383M and T386S are absent from 1000 genomes and gnomAD. Variant V382M appears 16 times in gnomAD and Y394S is present one time in 1000 genomes and 174 times in gnomAD.

**Table 3:** Activating variants in public databases and transcriptional activity.^a,b,c^

Nucleotide change	Protein change	Variant ID (rs#)	1000 genomes	gnomAD	Transactivation activity^c^
c.1144G>A	p.V382M	371918069	–	1 EAS 9 EUR 6 SAS (1 homozygote) Allele frequency: 16/282840 (0.00005657)	2.06**
c.1149C>G	p.I383M	–	–	^ –^	
c.1156T>A	pT386S	–	–	–	
c.1181A>C	p.Y394S	142287570	1 EUR (TSN) Allele frequency: 1/5008 (0.000199680)	134 Ashkenazi Jewish (2 homozygotes) 33 EUR 1 AFR 1 Latino 5 Other Allele frequency: 174/282840 (0.0006151)	2.38**

^a^Reference sequences: NM_004752.4 (transcript), NP_004743.1 (protein); ^b^No entry in ClinVar and HGMD (The Human Gene Mutation Database); ^c^fold over WT, data from GBS-luciferase assay, measured in Guan *et al.* (24). (**, *P* <0.01).

AFR, African, African–American; EAS, East Asian; gnomAD, Genome Aggregation Database; SAS, South Asian; EUR (European non-Finnish), TSN, Tuscany, Italy.

#### Analyses of the function of *GCM2* variants

Western blot analysis of extracts from HEK293-transfected cells showed that all variants associated with hyperparathyroidism had the same level of expression as WT GCM2 (Fig. 5C).

**Figure 6:**
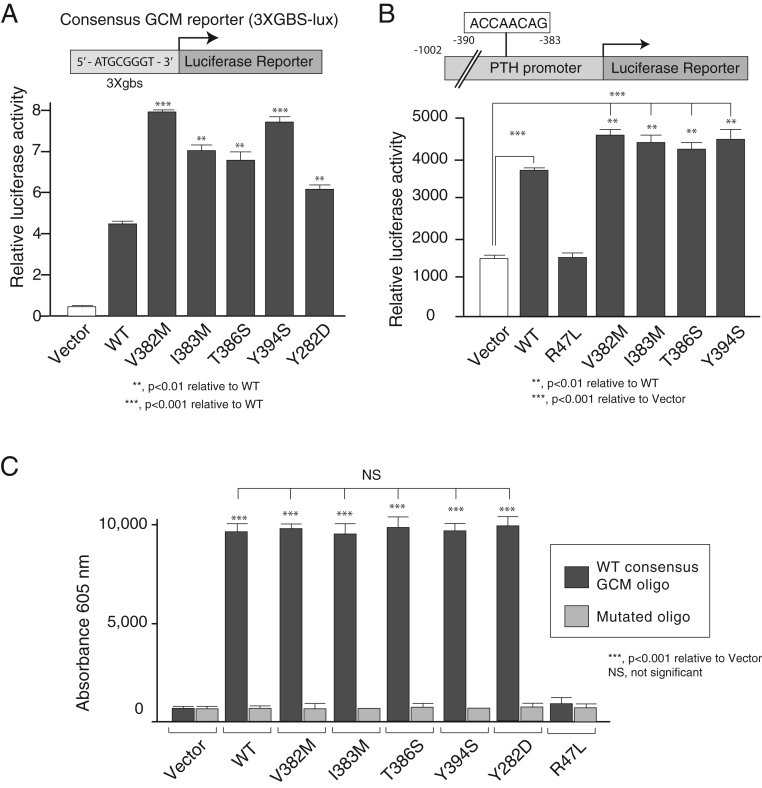
Transcriptional activities of WT and variant GCM2 proteins with a synthetic (3X-gbs) and natural (PTH) GCM2 responsive promoter. (A) A synthetic promoter–reporter construct containing three GCM response elements in tandem upstream of a luciferase reporter gene (3xgbs-lux) was transfected into HEK293 cells with vector, WT or variants (V382M, I383M, T386S, Y384S or Y282D). After 48 h, cells were harvested and luciferase activity of extracts measured. Values are mean ± s.e.m. of six estimations. (B) Top panel: representation of the human PTH promoter. Two putative GCM binding sites (−1002/−995) (site A) and (−390/−383) (site B) have been identified (41). Lower panel: the PTH promoter was transfected into HEK293 cells with vector, WT or variants (V382M, I383M, T386S, Y384S or Y282D). After 48 h, cells were harvested and luciferase activity of extracts measured. Values are mean ± s.e.m. of six estimations. (C) All activating variants bind DNA equally. Biotinylated oligonucleotide pull-down assays were conducted on extracts of HEK293 cells that had been transfected 48 h before harvesting with either empty vector alone or Flag-GCM2 WT or variants. The oligonucleotides used represented the WT and variant GCM consensus elements.

To assess their transcriptional activity, WT and mutant GCM2 constructs were co-transfected with the 3xgbs-lux reporter construct. Synthetic 3xgbs-lux exhibited basal activity when transfected with control pcMV-tag2. Co-transfection with WT GCM2 elicited a 4.3-fold increase in transcriptional activity relative to empty vector (Fig. 6A). The four variants and the previously described Y282D (32) all displayed significantly increased transcriptional activity relative to that of WT. GCM2 mutants V382M, I383M, T386S, Y394S and Y282D activated the 3Xgbs-lux activity 8-, 6.5-, 6.25-, 7.7- and 5-fold, respectively, over the empty vector control, representing 1.9, 1.5, 1.45, 1.8 and 1.2 times higher activity, respectively, than WT GCM2. The potency of our variants V382M and Y394S for transactivation of 3XGBS-lux was less than the one reported by Guan *et al*. (24) (Table 2). These discrepancies might be due to the use of a different synthetic promoter (6XGBS-lux vs 3XGBS-lux) and a different cell line (HEK293FT vs HEK293).

We also tested the ability of our variants to transactivate the PTH promoter. As shown in Fig. 6B, all the activating variants demonstrated transcriptional activity about 1.5 times higher that WT.

To assess if these variants would have an increase in DNA-binding which could explain their overactivity, we performed oligonucleotide precipitation assays with WT or mutated consensus *GCM* oligonucleotides (Fig. 6C). Equal binding of GCM2 WT and all variants proteins to the consensus oligo (but not the mutated one) was detected. In contrast, transcriptionally inactive control R47L did not bind to the *GCM* consensus oligo.

## Discussion

GCM2 has a fundamental role in fetal parathyroid gland development (12). Several gene mutations have been shown to cause various forms of familial isolated hypoparathyroidism, and while mutations in *preproPTH* and *CASR* genes affect PTH secretion, patients with homozygous inactivating *GCM2*mutations or heterozygous dominant-negative *GCM2* mutations develop hypoparathyroidism, presumably from parathyroid dysgenesis (15, 16, 19).

In the present study, we have identified a novel homozygous inactivating mutation of the *GCM2* gene in two families, with the affected individuals presenting in childhood with severe, symptomatic hypocalcemia and have provided biochemical evidence for a novel mechanism whereby it is causative of hypoparathyroidism.

The mutation, a substitution of arginine for cysteine at position 67 in the DBD of the transcription factor GCM2 (R67C), segregated with hypoparathyroidism in the families. The residue arginine 67 is conserved among species from human to *Drosophila*, suggesting its crucial role in GCM2 action (Fig. 1C). The R67C mutation was not present in the databases consulted in this study (Table 1) but two other variants, R67H and R67S, have been reported and we also tested their effect on GCM2 activity.

Our *in vitro* functional studies of GCM2 were performed in human embryonic kidney (HEK293) cells. These cells have been commonly employed (24, 34) but maybe a potential limitation of our functional studies in that we did not perform them in parathyroid cells; however, a stable human parathyroid cell line is not available. Thus, to date, studies have reported functional analysis (expression and/or activity) of GCM2 in cell lines such as, HEK293FT (24, 35), rat pituitary GH4C1 cells (16), African green monkey COS cells (42, 43) and chicken fibroblast DF-1 cells (44, 45), in addition to HEK293 cells (32, 34). HEK293 cells are human epithelial cells and thus seemed relevant for us to use in addition to having a high transfection efficiency, high reproducibility and having been shown to faithfully translate and process proteins accurately. Nevertheless, the parathyroid niche could potentially provide specific transcriptional co-regulators which have been shown to bind to GCM2 as well as other transcription factors which may still be unknown.

We provided novel insight into the structure−function relationships of the GCM2 mutant proteins that we identified in hypoparathyroidism, through several approaches. We first performed luciferase functional assay of the R67C GCM2 mutants found in FIH in HEK293 cells and unequivocally showed loss of transactivation function in co-transfection experiments using a synthetic consensus GCM-responsive promoter (3XGBS-Lux), despite unaltered protein expression as assessed by Western blot analysis. The variant, R67H, was able to transactivate the 3XGBS-lux as well as WT GCM2; however, R67S was as inactive as R67C. Oligonucleotide pull-down assays demonstrated that in contrast to WT and R67H, the transcriptionally inactive R67C and R67S variants had lost the ability to bind the consensus GCM recognition sequence.

The DBDs of mouse GCM1 and human GCM2 are 68% identical and 85% similar. Therefore, based on the published structure of the GCM1 DBD (38), we generated a human GCM2 DBD model to study the effects of the different variants. Arginine and histidine residues are polar positively charged amino acids that play a significant role in DNA-binding. In their 3D crystal structure, Cohen *et al.* (38) have determined that the polar residues of the GCM1 DBD form hydrogen bonds with the phosphate groups of some nucleotides of the consensus GCM consensus octamer. This contact is made by amino acids Arg62, Ser69, Lys73 and Lys74. We performed alignment and 3D comparative homology modeling of the human GCM2 DBD and identified human GCM2 arginine 67 as corresponding to murine GCM1 arginine 62 (Fig. 4A and  B). In contrast to arginine and histidine, cysteine and serine are polar non-charged amino acids. Consequently, R67C and R67S mutants are unable to bind DNA in our oligonucleotide precipitation assay and therefore showed no transcriptional activity in our luciferase assay. Substitution of histidine for arginine retains the positive charge at position 67, explaining why variant R67H is able to bind DNA and transactivate 3xGBS-lux as efficiently as WT GCM2. Taken together, our results provide novel insight into the mechanism whereby the R67C mutation is responsible for the autosomal recessive hypoparathyroidism in our two kindreds.

In the present study, we also identified heterozygous novel and recurrent variants of *GCM2* in the probands of 3 of 19 FIHP kindreds that were negative for mutations in the *MEN1*, *CDKN1B, CDC73*, *RET* or *CASR* genes. These variants are located in the CCID of GCM2 (amino acids 379–395) (24).

GCM2 has been reported to be downregulated, unchanged or upregulated in human parathyroid adenomas (30, 36). Although variant V382M has previously been reported in sporadic HPTH, when transiently transfected into chicken DF-1 fibroblasts, no differences between WT and variant GCMB were found in expression level, transactivation capacity and DNA-binding ability (31). Nevertheless, previous studies (24, 33, 35) identified other germline *GCM2* variants with increased *in vitro* transcriptional activity. Although, overactive or overexpressed mutated forms of *GCM2* could play a role in the evolution of parathyroid hyperactivity, to date, overexpressed mutated forms of GCM2 have not been reported in the literature.

In characterizing the biological action of the GCM2 variants we identified, we first found equal DNA-binding capacity using oligonucleotide pull-down assays. This likely reflects the independent actions of the GCM N-terminal DBD and the C-terminal domain (8). Our Western blot analyses showed that the protein basal level of expression of GCM2 variants is comparable, suggesting that the observed increase in transcriptional activities of CCID GCM2 variants is not due to increased protein stability but rather due to enhanced transactivating activity.

Previous studies have shown that the PTH promoter contains consensus GCM-binding sites and that WT GCM2 activates the PTH promoter in reporter assays (41). Our *in vitro* experiments indicate that GCM2 hyperactive variants display enhanced transactivation of the human PTH promoter compared to WT, which might possibly contribute to the development of hyperparathyroidism. It is possible that the activation of PTH transcription by GCM2 in parathyroid cells is by associating with other transcription factors such as GATA3 and MAFB (a known transcriptional regulator of parathyroid development), or other germline or somatic variants, and synergistically stimulating the PTH promoter (46, 47). Thus, the enhanced transcriptional activity of the CCID variants could be caused by a structural change allowing increased interaction with a transcriptional partner or reduced interaction with an inhibitory protein. In view of the limitation of using a non-parathyroid cell for our functional studies, the elucidation of these mechanisms awaits further study.

Although parathyroid tumor cells were reported to generally express less PTH mRNA than normal in some studies (48), no genetic analyses were reported in those studies and it is unclear which, if any, adenomas were familial. Furthermore, increased PTH mRNA expression was found in 1% of adenomas in that series and in parathyroid carcinomas and atypical adenomas. This is consistent with our finding of expression of variant GCM2 in both familial and sporadic parathyroid carcinoma with increased ability to stimulate PTH transcription. In other studies, PTH mRNA was reported to be increased in human adenomas relative to normal glands, PTH content was decreased and circulating (serum) PTH was increased (49) These findings are compatible with augmented newly synthesized PTH, minimal storage (50) and rapid secretion. Whether GCM2 variant-induced increased PTH mRNA expression, if present, could then cause increased PTH secretion and potentially contribute to hypercalcemia is possible but remains an area for future studies.

Genetic deletion of Gcm2 in mice displayed ‘shrinkage’ of the parathyroid glands, fewer parathyroid cells and a significant decrease in Casr- and Pth-expressing cells (51). Thus, a reduction of Gcm2 expression and transcriptional activity leads to a reduction of parathyroid cell proliferation, an increase in cell death and attenuation of parathyroid function. Consequently, it is possible that increased transcriptional activity of a GCM2 variant has the opposite effect and could contribute to parathyroid tumorigenesis as well as increased PTH production, but this will have to be assessed in future studies.

Parathyroid carcinoma is the rarest endocrine cancer accounts for 0.5–5% of all cases of PHPT (52, 53, 54, 55, 56, 57, 58, 59, 60, 61, 62). PC poses a diagnostic challenge because of the absence of characteristics that allow definite distinction of malignant from benign disease; however, capsular and vascular invasion have been linked with tumor recurrences and distant metastases and are considered the sole pathognomonic markers of malignancy (63, 64). PC may be sporadic or occur in the context of a genetic endocrine syndrome. Thus, PC may occur in 15% of individuals with HPT-JT (62, 65, 66), has rarely been reported in MEN2A syndrome (67) and is infrequent (0.28–1%) in MEN 1 (68, 69). The most common genetic anomalies associated with PC are inactivating somatic mutations of the parafibromin gene (*CDC73/HRPT2*). PC may also occur in 1% of FIHP patients (70, 71, 72). A GCM2-PC association has previously been suggested (35, 73), but unavailability of a rigorous pathological diagnosis may have limited these observations. We report here a heterozygous *GCM2* variant in a subject with PC in one of our three FIHP families and also identified a heterozygous germline variant of *GCM2* in one of sixteen subjects with apparently sporadic PC that was negative for mutations in the *CDC73* gene. Both had strong histopathological evidence of parathyroid cancer. Consequently, whether identification of germline *GCM2* variants may sometimes contribute to a more malignant form of hyperparathyroidism requires further consideration.

In conclusion, we have identified novel and recurrent heterozygous activating variants of the *GCM2* gene and provided evidence that they are potential contributors to the pathogenesis of hyperparathyroidism and could be associated with parathyroid carcinoma. Although our families are relatively small and predictive values are as yet unknown, our studies increase the likelihood that scoring these variants will eventually have a role in patient management and for screening of families and may also point to potentially more aggressive nature of parathyroid disorders associated with activating GCM2 variants. Nevertheless, although we hypothesize that GCM2 variants can play an important role in parathyroid physiology and pathophysiology, they likely need to cooperate with other factors. Future studies aimed at identifying co-operating factors that may help in transmitting the actions of GCM2 may therefore further delineate the potential role of variant GCM2 in causing parathyroid tumors and biochemical hyperparathyroidism.

## Supplementary Material

Supplementary Table 1 Characteristics of FIHP Kindreds

Supplementary Table 2 Characteristics of Parathyroid Carcinoma Patients

## Declaration of interest

The authors declare that there is no conflict of interest that could be perceived as prejudicing the impartiality of the research reported.

## Funding

This work was supported by a grant from the Canadian Institutes of Health Researchhttp://dx.doi.org/10.13039/501100000024 to D G, Funds RC2018-2019-2020 from Italian Health Ministry to A S, and by a very generous donation to the Research Institute-McGill University http://dx.doi.org/10.13039/100008582 Health Centre from Jennifer Reid in memory of Dr G N Hendy.

## Data availability

All data generated or analyzed during this study are included in this published article or in the data repositories listed in references.

## Author contribution statement

L C and D G conceived and designed the experiments. L C, V G, Y K, B W and A N-L performed the experiments. V G, D E C C, S M, C E-V, F C, A R, D T, S C and A S provided essential study material. L C, V G, Y K, B W and A S analyzed the data. L C and D G wrote the manuscript. All authors read, revised and approved the submitted version of the manuscript.
