# Supplementary material for: Novel Glial Cells Missing-2 (GCM2) variants in parathyroid disorders
Source: Eur J Endocrinol. 2022 Jan 13;186(3):351–66. doi: 10.1530/EJE-21-0433 (PMC8859918; doi:10.1530/EJE-21-0433)
Supplement: Supplementary Table 1 Characteristics of FIHP Kindreds [file supplementary_table_1.pdf]

Supplementary Table 1 Characteristics of FIHP Kindreds																					
Characteristics of 19 Kindreds Studied									Initial Chemistries (normal range)						Parathyroid Characteristics				Clinical Manifestations		
Pedigree Location	Family 1,2 0r 3 of Fig 5A	Kindred Identifier	[a]No	Relation to Proband	Pedigree identifiers	Sex	GCM2 Variant	[c]Age at DX of PHPT (Y)	[d] Ser Tot Ca (2.05-2.50 mmol/L)	Ser Int PTH (10-65 pg/mL)	Ser Ion Ca^{2+} (1.17-1.31 mmol/L)	Ser P (2.7 - 4.5 mg/dL)	24-h Urine Ca (1.3 - 7.5 mmol/24 h)	[f]UCCR	Tot No of Excised Glands	Normal Glands by Biopsy (y/n)	Largest Size of Largest Gland (cm)	Pathology of Excised Parathyroids	Osteoporosis (O) Nephrolithiasis (N) Fractures (F) Yes (y)/No (n)		
																			O	N	F
San Giovanni Rotondo	2	VS	6	proband	II:3	M	[#]p.T386S (het)	58	3.7	113	2.25	2.2	36.7	0.023	3	n	1.8	hyperplasia	y	y	n
				mother	I:2	F	p.T386S (het)	90*	2.23	62	[e]na	3.7	na	na	/	/	/		na	y	na
				son	III:1	M	p.T386S (het)	29*	2.4	58	1.22	3.2	na	na	/	/	/		na	y	na
				sister	II:2	F	p.T386S (het)	65	2.99	104	1.56	2.9	na	na	4	n	2.6	hyperplasia	n	y	n
				sister	II:1	F	[b]Neg	69*	2,27	56	na	3,2	na	na	/	/	/		na	n	na
				brother		M	Neg	67*	2.37	51	na	na	na	na	/	/	/		na	n	na
		NP W	2	proband		F	Neg	59	2.9	76	1.45	3.21	5.7	0.016	2	y (1)	1	adenoma	y	y	n
				sister		F	Neg	60	2.62	82	1.31	2.45	6.4	0.021	1	n	0.8	adenoma	y	y	n
		OS	2	proband		F	Neg	35	3.75	1410	1.89	2.4	13.6	0.056	1	n	3	adenoma	n	n	n

				mother		F	Neg		2.92	118	1.48	2.58	5.2	0.02	1	n	1	hyperplasia	n	n	n
		NR	2	proband		F	Neg	47	2.96	251	1.49	1.63	10.7	0.032	3	n	1.5	hyperplasia	y	y	n
				sister		F	Neg	19	2.74	61	1.38	3.84	4.8	0.012	/	/	/		n	y	n
		BF	2	proband		F	Neg	40	2.8	155	1.41	2.5	10.1	0.03	1	n	1.2	adenoma	y	n	y
				mother		F	Neg	63	2.74	112	1.36	3.47	4.2	0.012	2	n	1	hyperplasia	y	n	y
Rome		SV	2	proband		F	Neg	51	2.65	37	1.40	4.3	7.97	0.019	/	/	/		y	y	n
				sister		F	Neg	53	2.68	43	1.37	3.15	4.98	0.016	/	/	/		y	n	n
		CT	3	proband		F	Neg	69	2.82	112	1.41	2.6	9.12	0.023	1	n	1.2	hyperplasia	y	y	n
				daughter		F	Neg	45	2.78	163	1.34	2.9	7.42	0.02	/	/	/		n	n	n
				nephew		M	Neg	40	2.75	86.5	1.39	2.5	7.22	0.02	/	/	/		n	n	n
Milan		FD	2	proband		M	Neg	77	3.1	173	1.56	1.6	9.7	na	refused surgery	/	/		Y	n	y
				son		M	Neg	55	2.75	174	1.39	1.7	12.7	na	1	n	1.7	adenoma	Y	y	y
		CG	3	proband		F	p.Y282D (het)	49	2.52	86.4	1.34	2.96	7.7	na	3	n	1.4	adenoma hyperplasia	n	y	n
				daughter		F	Neg	34	2.6	114	1.38	4.2	10.5	na	3	n	1.6	hyperplasia	n	n	n
				daughter		F	Neg	28	2.76	135	1.54	1.8	13.9	na	1	n	1	adenoma	n	n	n
	1	PG	3	proband	I:2	F	**p.Y394S (het)**	62	2.62	62	1.37	3.2	7.1	0.023	[§]No surgery yet	/	/		y	n	n
				son	II:1	M	**p.Y394S (het)**	44	2.65	43.2	1.33	2.9	4.6	na	/	/	/		n	n	n
				son	II:2	M	**p.Y394S (het)**	43	2.55	33.5	1.32	2.83	5.6	0.02	/	/	/		n	y	n
Pisa		296	2	proband		F	Neg	37	na	161	1.67	na	na	na	1	n	na	adenoma	na	na	n
				daughter		F	Neg	20	2.62	39	1.32	2.7	2.62	0.019	1	n	1.1	adenoma	n	n	n
		349	2	proband		F	p.Y282D (het)	56	2.42	81	1.36	3	8.6	0.025	3	n	1.2	hyperplasia	Y	y	n
				sister		F	Neg	57	2.74	85	1.38	3	2.8	0.0095	/	/	/		Y	n	n

		671	2	proband		F	Neg	59	3.17	640	1.8	2.1	3.47	0.01	1	n	4	carcinoma	Y	y	n
				mother		F	Neg	69	na	na	na	na	na	na	1	n	1.8	adenoma	na	na	na
		799	2	proband		M	Neg	22	3.77	252	2.04	2.5	11.1	0.027	1	n	3	adenoma	Y	y	n
				mother		F	Neg	48	2.57	66	1.39	2.1	10.2	0.02	1	n	1.4	adenoma	n	n	n
		388	2	proband		F	Neg	53	2.92	252	1.53	2.3	5.37	0.02	3	n	3.0	hyperplasia	Y	n	n
				daugher		F	Neg	25	na	59	1.35	na	6.72	na	/	/	/		n	n	n
		503	2	proband		F	Neg	33	2.4	55	1.35	3.5	3.63	0.012	1	n	1		n	y	n
				father		M	p.Y282D (het)	60	2.69	248	1.53	2.6	5.95	na	1	n	1.5	adenoma	Y	y	n
		573	2	proband		F	Neg	43	2.6	143	1.36	3.2	12.22	0.027	1	n	1.4	adenoma	n	y	n
				mother		F	Neg	67	2.52	88	1.39	3.3	4.4	0.017	/	/	/		y	n	n

Symbols:

#Novel variants identified in kindreds in this study are denoted in red

* age at recruitment

		823	2	proband		F	Neg	48	2.57	226	1.5	2.75	8.4	0.01	1	n	2.3	adenoma	Y	y	n
				daughter		F	Neg	18	2.71	81	1.41	2.3	4.7	0.01	1	n	1.8	adenoma	n	n	n
Bologna	3	GI	4	proband	II:1	M	**p.I383M (het)**	38	3.29	230	na	2	13.97	na	3	n	3.5	carcinoma hyperplasia	n	y	n
				mother	I:2	F	**p.I383M (het)**	67	2.57	95	na	2.6	5.34	na	2	n	1	adenoma	y	n	y
				uncle	I:3	M	**p.I383M (het)**	55	3.24	841	na	1.9	5.49	na	3	n	4.5	adenomas atypical adenoma	y	n	y
				sister	II:1	F	Neg	42*	2.1	30	na	2.76	na	na	/	/	/		n	n	n

§ Neck ultrasonography: two extrathyroidal nodules behind the right and left thyroid lobes, suggestive of parathyroid lesions

Superscripts:

[a] No=number studied per kindred

[b] Neg=Tested negative for gcm2 variants

[c] Age at DX of PHPT (Y) = Age at Diagnosis of PHPT in years (Y) [d] Ser Total Ca =Serum Total Calcium, albumin-adjusted [e]

na=not available

[f] UCCR=Urinary Ca/Creatinine Clearance Ratio
