# Supplementary material for: Novel Glial Cells Missing-2 (GCM2) variants in parathyroid disorders
Source: Eur J Endocrinol. 2022 Jan 13;186(3):351–66. doi: 10.1530/EJE-21-0433 (PMC8859918; doi:10.1530/EJE-21-0433)
Supplement: Supplementary Table 2 Characteristics of Parathyroid Carcinoma Patients [file supplementary_table_2.pdf]

Supplementary Table 2<br>Characteristics of Parathyroid Carcinoma Patients																
Patient Characteristics				Initial Chemistries (normal range)						Parathyroid Characteristics				Clinical Manifestations		
Patient Name	Sex	GCM2 Variant	[a] Age at DX of PHPT (Y)	[b] Ser Total Ca (2.05-2.50 mmol/L)	Ser Intact PTH (10-65 pg/mL)	Ser Ion Ca^{2+} (1.17-1.31 mmol/L)	Ser P (2.7 - 4.5 mg/dL)	24-h Urine Ca (1.3 - 7.5 mmol/24 h)	[d]UCCR	Total [e]no of Excised Glands	Normal Glands by Biopsy (y/n) (no)	Largest Size of Largest Gland (cm)	Pathology of Excised parathyroids	Osteoporosis (O) Nephrolithiasis (N) Fractures (F)<br><br>Yes (y)/ No (n)<br><br>O	N	F
PA	F	**p.(V382M) (het)**	49	5.0	940	2.5	1.34	na	na	1	n	4	carcinoma	n	n	n
KA	F	None	55	3.87	873	2.35	2.2	19	0.225	2	y (1)	3	carcinoma	na	y	na
BM	M	None	76	3.37	1052	2.13	1.82	9.3	0.056	4	n	4.5	carcinoma + atypical adenoma	n	y	n
CN	F	None	65	3.32	229	[c]na	2.2	na	na	1	n	4	carcinoma	y	n	n
CM	M	None	44	2.52	163	1.38	2.6	na	na	1	n	2	carcinoma	y	na	n
CA	M	None	57	3.72	690	2.13	2.5	9.7	0.045	1	n	3.5	carcinoma	n	n	n
GA	M	None	57	3.22	1791	na	1.5	na	na	4	y (3)	3	carcinoma	y	y	y
LA	F	None	69	3.45	852	1.91	1.72	5.6	0.022	1	n	2.9	carcinoma	n	n	n
MM	F	None	48	3.79	525	na	2.2	na	na	1	n	7	carcinoma	na	y	na
OR	F	None	43	3.09	344	na	na	na	na	2	y (1)	5	carcinoma	n	y	n
PN	F	None	77	3.04	536	na	2.39	11.15	na	2	y (1)	2	carcinoma	y	y	n
PAM	F	None	46	3.09	539	1.57	1.84	na	na	3	y (1)	2.5	carcinoma	y	y	n
PM	F	None	53	4.52	2665	2.31	na	na	na	2	y (1)	3.8	carcinoma	y	n	n
RMV	F	None	58	2.92	642	na	na	13.4	na	2	y (1)	6	carcinoma	y	na	y
TA	F	None	34	3.06	204	1.62	2.54	2.48	0.039	1	n	1.3	carcinoma	n	y	n
TR	M	None	55	3.22	252	1.54	2.48	14.3	na	1	n	4.1	carcinoma	n	y	n

Superscripts:

[a] Age at DX of PHPT (Y)=Age at Diagnosis of PHPT in years(Y)

[b] Ser Total Ca =Serum Total Calcium, albumin-adjusted [c]

na=not available [d] UCCR=Urinary Ca/Creatinine Clearance

Ratio

[e] no=number
